# A Case of Isolated Cervicomedullary Injury With Extensive Wallerian Degeneration: The Multifactorial Nature in Making Decisions to Withdraw Life Support From a Pathology Perspective

**DOI:** 10.7759/cureus.104542

**Published:** 2026-03-02

**Authors:** Jiayi Li, Steven N Schwartz

**Affiliations:** 1 Pathology and Laboratory Medicine, Thomas Jefferson University Hospital, Philadelphia, USA

**Keywords:** forensic pathology, neurology, neuropathology, traumatic spinal cord injury, withdrawl of life support

## Abstract

Patients suffering from traumatic spinal cord injury (SCI) constitute a unique patient population in critical care medicine due to the high rate of mortality, irrecoverable neurological damage, and dependence on advanced life support measures. In this report, we present a patient who sustained a non-survivable cervicomedullary injury due to motor vehicle collision who became dependent on maximal life support and subsequently expired from multiple complications 11 months after the initial accident. On neuropathological exam, extensive Wallerian degeneration in the spinal cord despite non-traumatic findings in bilateral cortices corroborate the clinical picture. We report in detail the clinical presentation, hospital course, and autopsy findings surrounding this case. Additionally, we discuss the socioeconomic factors, medical complexities, and regulatory guidelines surrounding the decision to withdraw life support when prognoses are guarded.

## Introduction

In the U.S., over 12,000 patients suffer from traumatic spinal cord injury (SCI) every year [1]. Of these injuries, complete cervical SCI involving odontoid fractures is highly lethal and non-recoverable due to severe neurologic sequelae such as quadriplegia and loss of diaphragmatic function [2]. The American Spinal Injury Association Impairment Scale (AIS) defines SCI Scale A as the sustained injury being complete impairment without residual motor and sensory function below the level of injury. However, the conversion of AIS to clinical prognosis and mortality rate is not clearly defined. Often, the prediction of individual patient outcome from imaging results (e.g. CT, MRI) alone is conveyed to the health-decision makers of the patient in making decisions to withdraw life-supporting treatment (WLST). The exact injury pattern and implication can hardly be elucidated without postmortem pathology examination. The key pathophysiology contributing to the severe sequalae of SCI is Wallerian Degeneration. This is a well-known phenomenon when irreversible neuronal death occurs rapidly after the neuronal axons are severed from the cell body. Meanwhile, the cost of care in the first year after initial injury can accrue to upwards of $1,156,410 for AIS A, B, and C [3]. In addition, the medical complications associated with prolonged hospital stay further exacerbate the patient’s quality of life. The factors associated with whether or when the decision to withdraw life support is made include age, sex, race, history of dementia, Glasgow Coma Scale (GCS) score, SCI level, and concurrent injuries to head or thorax [4]. In the following case, we would like to demonstrate the unique role of pathology in unpacking the complex interplay of these factors and the importance of educating and communicating with the family to make the most appropriate decision for the patient.

## Case presentation

Initial presentation

The decedent was an 80-year-old man with a past medical history notable for cerebrovascular accident, end-stage renal disease, atrial fibrillation, hypertension, anemia, alcohol and cocaine use disorders. He initially presented with GCS of 3 on arrival after a motor vehicle collision with pulseless electrical activity arrest in the field but return of spontaneous circulation (ROSC) was achieved within five minutes. He was also found to have a severe C2 SCI (AIS A), accompanied by an extensive hemorrhagic contusion of the brainstem and spinal cord extending from C3 to the mid-medulla. Additional findings included subarachnoid hemorrhage with intraventricular extension which was managed with right frontal burr hole for decompression, chronic ununited type 2 odontoid fracture with destabilizing ligamentous disruption and left frontal scalp contusion. Neurosurgery was consulted and no surgical interventions were offered due to the C2 dens fracture representing a non-recoverable and non-survivable injury pattern. A continuous electroencephalography (cEEG) was performed on day 3 of admission, which showed continuous background theta and delta wave activity with no seizures. GCS remained at 3, and the patient was noted to have facial movement without motor response to painful stimuli in bilateral upper or lower extremities. According to records, the patient's family was aware of the patient's poor neurologic prognosis and elected for ongoing aggressive care, including tracheostomy and percutaneous endoscopic gastrostomy (PEG), which were performed on day 15 of admission. The patient remained ventilator-dependent thereafter. GCS improved to 6 (eye 4, verbal 1, motor 1) and remained stable at this point. 

Evolving injuries and clinical complications

**Figure 1:**
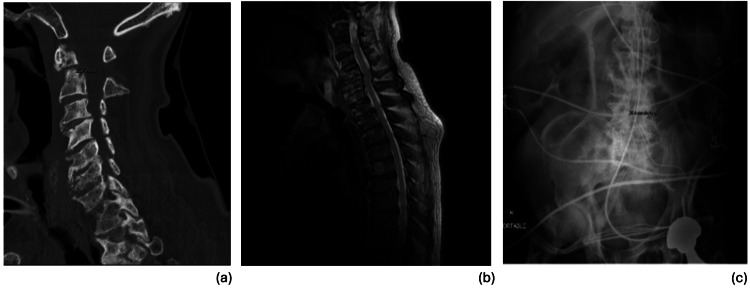
Significant radiological findings during hospital course (a) CT cervical spine demonstrates Type II fracture of the C2 vertebra with approximately 2.7 mm of superior distraction of the dens fracture fragment; (b) MRI cervical spine demonstrates fluid cleft at the known non-united type II odontoid fracture. There is abnormal cord signal at the cervicomedullary junction, with small rim-enhancing area, representing evolving hemorrhagic contusions; (c) Abdomen X-Ray demonstrates questionable short segment of large bowel with featureless wall in the mid abdomen, which can be seen in the setting of ischemia.

On day 21 of admission, he was noted to have altered mental status. Prognosis-defining radiological findings are represented in Figure 1. A CT angiography (CTA) A head and neck was obtained, which revealed new displacement of the superior fragment of the type 2 odontoid fracture, with rightward offset of C1 on C2 lateral mass bilaterally (Figure 1a). An MRI brain showed an acute infarct in the cervicomedullary junction (Figure 1b). His hospital course was further complicated by atrial arrhythmias, ventilator-dependent respiratory failure, vasopressor-requiring shock, renal failure with initiation of intermittent hemodialysis, leukocytosis, and respiratory infections with Serratia and Pseudomonas species.

The patient was transferred to Neuro Critical Care on day 47 of admission for continued management involving numerous infectious and systemic complications. His GCS subsequently improved and stabilized to 8 with spontaneous eye-opening to voice at times and withdrawing to pain until the end of the year. Occasional lip smacking and intermittent spasms on deep palpation of abdomen and lower extremity were noted by the care team. Around day 210 of hospitalization, his baseline GCS declined to 5 (sub score 3-1-1) with eye-opening to voice without following command.

Multiorgan failure and death

**Figure 2:**
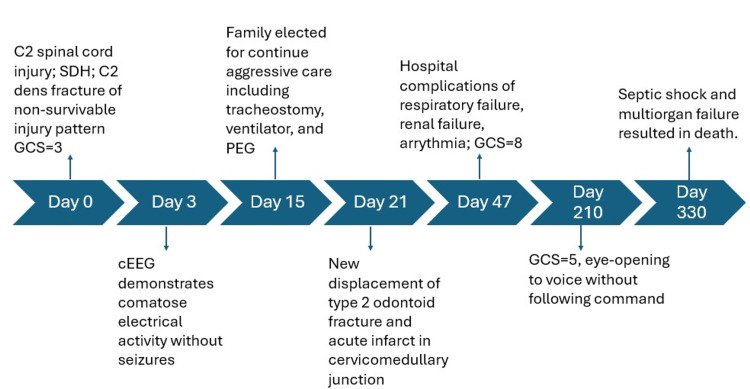
Timeline summarizing radiological findings and clinical events SDH: Subdural hematoma; GCS: Glasgow Coma Scale; cEEG: Continuous electroencephalography; PEG: Percutaneous endoscopic gastrostomy

Around day 330 of hospitalization, the patient was noted to have increased pressor requirements. Stress dose steroids were added, and a bicarbonate drip was initiated. The family was called urgently, and a desire to continue full restorative measures was reiterated. Notably, there was concern for gastrointestinal bleed, septic shock, and hypovolemic shock in the setting of significant lactic acidosis. An abdominal X-ray was obtained with findings suggestive of possible ischemic bowel (Figure 1c). His labs were notable for worsening mixed acidosis, thrombocytopenia, anemia, and coagulopathy (Table 1). The patient was further transfused and dialyzed yet continued to have worsening oxygen saturations despite maximal ventilator settings. Following extensive discussions regarding the patient's condition and goals of care, the patient's family expressed the desire for the patient to remain full code. Despite maximal supportive measures, multiorgan failure progressed with worsening of the patient's respiratory failure and declining blood pressure. Cardiopulmonary resuscitation was initiated but was unsuccessful. The patient was pronounced dead the day after. Autopsy revealed the immediate cause of death to be sepsis with extensive bilateral pneumonia complicated by circulatory and multiorgan failure due to prolonged hospitalization due to traumatic SCI. The manner of death was accident. A timeline summarizing key radiographical findings and clinical events is presented in Figure 2. 

**Table 1:** Critical lab values PT: Prothrombin time; INR: International normalized ratio; PTT: Partial thromboplastin time

​Laboratory Tests	Patient Value ​	Reference Value ​
Arterial pH​	6.94 ​	7.35-7.45 ​
Platelet ​	71 B/L​	140-400 B/L​
PT​	45.5 s​	9.4-13.0 s​
INR​	4 s​	0.87-1.19 s​
PTT​	56 s​	25-37 s​
Lactate ​	15 mmol/L​	0.5-2.0 mmol/L​

**Figure 3:**
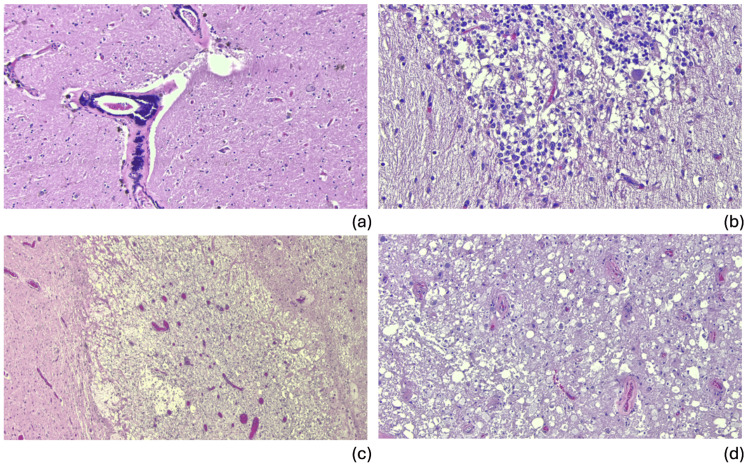
H&E photomicrographs of significant neuropathology findings (a) Hypertensive vascular changes and arteriolosclerosis in left basal ganglia; (b) Right cerebellum demonstrates Bergmann gliosis with Purkinje cell dropout consistent with hypoxic-ischemic injury; (c) Cervicomedullary junction showing subacute infarcts with associated Wallerian degeneration and histiocytosis; (d) Representative section of spinal cord demonstrating extensive Wallerian degeneration. H&E: Hematoxylin and eosin

Neuropathological examination demonstrated an adult brain weighing 1,230 g (normal range: 1,179-1,621 g in men). Both external and internal surfaces of the dural leaflets were smooth. There was one focal calcified nodule. The external surfaces of the brain were symmetric and exhibited no evidence of atrophy, softening, or discoloration. There was no evidence of herniation of the cingulate gyri, unci, or cerebellar tonsils. There was patchy leptomeningeal fibrosis. The arteries of the circle of Willis and their major branches were patent, with mild to moderate atherosclerosis. Serial coronal sections of the cerebral hemispheres revealed no gross abnormalities. Cross sections of medulla and cervical-medullary junction revealed granular and plaque-like changes implying underlying pathology. There was mild superior cerebellar vermal atrophy on the right. Microscopic examination demonstrated hypertensive vascular changes in right frontal cortex, left basal ganglia, and the midbrain (Figure 3a). The thalamus demonstrated diffuse vascular congestion. The anterior superior cerebellar vermis was atrophied, consistent with chronic alcohol use disorder. In addition, there was notable dropout of Purkinje cells with variable eosinophilia and Bergmann gliosis consistent with hypoxic-ischemic injury (Figure 3b). Cervicomedullary junction revealed subacute to chronic infarct with associated Wallerian degeneration (Figure 3c). The remainder of the spinal cord sections showed bilateral and extensive Wallerian degeneration (Figure 3d). 

## Discussion

This case is unique and thought-provoking, not only in the diverse presentation of post-SCI functional neurology but also the multifactorial clinical decision-making process in WLST pertaining to bioethical consideration, grief counseling, and patient education. We will address the stated areas in the context of our patient through a detailed chart review. 

The patient self-identified as non-White, retired, and religious. His marital status was divorced, and his primary health decision makers were his sons and daughters. The legal power of attorney and advanced healthcare directive was initially shared amongst the four adult children, then eventually given to his oldest son. The patient was unfortunately denied by all long-term acute care facilities due to ventilation dependence and lack of insurance authorization. Around two months prior to the date of death, out of respect for the family’s preference for plan of care, there was no further discussion on comfort care or code status. The clinical team provided only medical updates to the family. Furthermore, comfort care was not in line with the family's religious beliefs and under no circumstance would they want withdrawal from full medical intervention. According to Shakil et al., in a retrospective multicentric observational cohort study, Black and Asian patients are less willing to undergo WLST [4]. However, advanced age, male sex, White race, prior dementia, low GCS score, prior history of cardiac arrest, and high SCI level are associated with increased likelihood of WLST. The highly contested case of Jahi McMath involved a 13-year-old patient who underwent tonsillectomy and suffered from massive hemorrhage, which resulted in severe brain injury. Despite achieving ROSC, she was declared brain dead after three days [5]. As devout Christians, the family strongly objected to WLST and sued the hospital for unilateral withdrawal of ventilation. The patient was subsequently placed in a facility until she expired from liver failure five years later. Although the brain stem death (BSD) was never declared in this patient, the shared themes such as definition of death and the religious implication of WLST are resounding. The definition of death remains up for debate since the concept of whole-brain death in 1968 [6]. Legislatively, the Accommodations & Brain Death Act was enacted in 2009 as a result of this case. This clause allows for a “reasonably brief” period of time for accommodation (including ventilation and other medical support). Nevertheless, the actual application of this law varies largely on an individual basis.

Despite low GCS score and guarded EEG results (representative of comatose state), our patient never met the criteria of brain death [7,8]. Cognitive function assessment was limited, but according to clinical notes, it appears that the patient might have retained some level of cognition until day 58 of admission with attempt to verbalize but failed to demonstrate voice tracking or command following. It is unclear whether the persistent spontaneous eye-opening observed was due to auditory-ocular reflexes or any remaining cognitive function. The patient became ventilator-dependent soon after the new-onset cervicomedullary infarct demonstrated on MRI. Nevertheless, his ability to withdraw to pain in lower extremities and exhibit spasmatic muscle movement suggest the possibility of preserved conduction pathways from the lower spinal cord to the cortex, or could be attributable to hyperreflexia due to upper motor neuron lesion. Given his unique injury pattern and the functional consequences, it is beyond difficult to convince the family of WLST. According to Shaikh et al., in a systematic review of families’ experiences after receiving BSD diagnoses, the cognitive dissonance of being told the patients are brain-dead and seeing patients still warm and breathing appear to be the major barrier to accepting the diagnosis [9]. In contrast, in a randomized control trial, questionnaires sent to patient families demonstrated that those who took the opportunity to observe a BSD test scored higher in terms of their understanding of BSD compared to those who did not [10]. Furthermore, those who observed a BSD test reported lower levels of psychiatric distress compared to their usual state of mental health. This is a significant finding as it appears that increased understanding of BSD through test observation can help mitigate the process of grief experienced by patient families.

In this case, based on the clinical picture and neuropathological examination, it is reasonable to conclude that despite sustaining severe high-level SCI, the patient’s brain and spinal cord sustained no direct structural damage and were likely functional in isolation. This is further confirmed by neuropathology examination at the time of autopsy. Should the patient have suffered global ischemic injury to the cerebral cortex, we would expect to see a diffuse dusky discoloration of the cerebral cortex, blurring cortical-medullary junction and general friability of the brain [11]. Further, we would expect extensive cytolysis, autolysis, and possible leukclastic reaction microscopically. However, the structural integrity of the patient’s brain was well preserved, and there were no findings of cortical infarcts. As for the Bergmann gliosis occurs in response to localized, ischemic injury, resulting in cerebellar scarring. According to the patient’s history, this finding likely represents a remote cerebrovascular accident. Nevertheless, there was extensive Wallerian degeneration of axons observed in the spinal cord distal to the cervicomedullary infarct over the prolonged hospital course at the time of death. The histopathological remodeling in the cervicomedullary junction characterized by extensive histiocytosis also corroborates with timing of the evolving injury. In the original experiment of Augustus Waller, the axons in mouse nerve transection models did not survive past 1.5 days once severed from the cell bodies, and was characterized by granular disintegration microscopically [12]. Overall, these findings corroborates the clinical presentation of seemingly preserved cortical structure and function despite the diagnosis of a non-survivable injury mostly severing the connection between the brain and the spinal cord based on imaging. We also recognize that the inability to fully assess cognition or electrophysiologic conduction is a limitation in this case. Nevertheless, we hope the insights from neuropathology exam would make up the lack of functional testing in strengthening the rigor of clinicopathological correlation.

## Conclusions

In conclusion, this case demonstrates an example of a traumatic high-level SCI pattern that produced unique neurological functional outcomes. Neuropathological findings from autopsy were crucial in clarifying and explaining the clinical presentation of subacute incomplete cervicomedullary infarct with subsequent Wallerian degeneration. Additionally, the care decision on continuing advanced life support despite guarded prognosis increases the awareness of the importance of patient education, grief counseling, and legislative guidance on this subset of patient population.

## References

[1] (2014). Spinal cord injury facts and figures at a glance. J Spinal Cord Med.

[2] Aarabi B, Neal CJ, Hersh DS (2023). Mortality in ASIA Impairment Scale grade A to D patients with odontoid fracture and magnetic resonance imaging evidence of spinal cord injury. Neurotrauma Rep.

[3] Malekzadeh H, Golpayegani M, Ghodsi Z (2022). Direct cost of illness for spinal cord injury: a systematic review. Global Spine J.

[4] Shakil H, Malhotra AK, Jaffe RH (2023). Factors influencing withdrawal of life-supporting treatment in cervical spinal cord injury: a large multicenter observational cohort study. Crit Care.

[5] Friedrich AB (2019). More than “spending time with the body”: the role of a family’s grief in determinations of brain death. J Bioeth Inq.

[6] Johnson LS (2016). The case for reasonable accommodation of conscientious objections to declarations of brain death. J Bioeth Inq.

[7] Trinka E, Leitinger M (2015). Which EEG patterns in coma are nonconvulsive status epilepticus?. Epilepsy Behav.

[8] Greer DM, Kirschen MP, Lewis A (2023). Pediatric and adult brain death/death by neurologic criteria consensus guideline. Neurology.

[9] Shaikh M, Cade-Smith E, Mackay L, Wijayatilake DS, Kingsley M (2024). Brain stem death diagnosis: a systematic review of families’ experience. Discov Psychol.

[10] Tawil I, Brown LH, Comfort D (2014). Family presence during brain death evaluation: a randomized controlled trial*. Crit Care Med.

[11] Wijdicks EF (2024). The respirator brain: a reckoning. Neurocrit Care.

[12] Coleman MP, Freeman MR (2010). Wallerian degeneration, WldS, and Nmnat. Annu Rev Neurosci.

